# Automated irrigation systems in mitigating laser‐induced temperature during simulated ureteroscopy

**DOI:** 10.1111/bju.70364

**Published:** 2026-07-07

**Authors:** Richard Menzies‐Wilson, Jessica Williams, Candace Rhodes, Leilane Glienke, William W. Roberts, Ben Turney

**Affiliations:** ^1^ Nuffield Department of Surgery University of Oxford Oxford UK; ^2^ Boston Scientific Corporation Marlborough MA USA; ^3^ Department of Urology University of Michigan Ann Arbor MI USA

**Keywords:** ureteroscopy, laser, power, flow rate, temperature, fluid management system, FANS

## Abstract

**Objective:**

To compare the temperature changes with laser activation when providing irrigation with a pressure bag vs a fluid management system (FMS).

**Materials and Methods:**

Benchtop experiments were performed with a LithoVue™ Elite (Boston Scientific Corp., Marlborough, MA, USA) flexible ureteroscope placed through an 11/13‐F ureteric access sheath into a benchtop pelvicalyceal kidney model. Room temperature saline irrigation, via the scope, was provided either with a pressure bag or FMS. Both systems were pressurised to 200 mmHg and left for 10 min. After 10 min, a holmium:yttrium‐aluminium‐garnet laser was activated at 20, 40, or 60 W for 90 s. Flow rate, irrigation pressure, intrarenal pressure and calyceal temperature were recorded continuously. Experiments were repeated five times. Benchtop empirical temperature data were compared to mathematical model predictions. The mathematical model was then used to predict calyx temperature as a function of flow rate in *in vivo* conditions.

**Results:**

Pressure‐bag irrigation flow rate declined from ~38 to ~25 mL/min (36%) over 10 min, whereas the FMS maintained stable flow of ~45 mL/min. Intrarenal pressure remained ≤30 mmHg in all experiments. Consequently, the benchtop calyx mean (standard deviation) temperature rise with pressure bag vs FMS irrigation was: at 20 W 7.9 (1.0)°C vs 6.4 (0.6)°C (*P* = 0.02); at 40 W 12.8 (3.2)°C vs 10.3 (1.7)°C (*P* = 0.15); at 60 W 17 (3.4)°C vs 12.4 (1.2)°C (*P* = 0.02).

**Conclusion:**

Irrigation flow rate is a key determinant of thermal risk during laser lithotripsy. An automated FMS can maintain consistent flow; mitigating temperature rise and thermal dose in a benchtop model.

**Abbreviations**
CEM43: cumulative equivalent minutes at 43°C
eq min: equivalent minutes
FANS: flexible and navigable access sheaths
FMS: fluid management system
Ho:YAG: holmium:yttrium‐aluminium‐garnet
IRP: intrarenal pressure
LVE: LithoVue Elite
t43 equivalent: CEM43 thermal dose of laser activation
UAS: ureteric access sheath

## Introduction

Ureteroscopy and laser lithotripsy is the most commonly performed operation to treat renal stones [1]. Benchtop studies have suggested that increasing laser power may increase stone ablation speed (mm^3^/s), decreasing procedure time [2, 3, 4, 5, 6, 7, 8, 9, 10]. However, >90% of laser energy is converted to heat and so higher power laser activation – and in particular high pulse frequency – can raise renal temperatures proportionally, risking thermal injury [11, 12, 13, 14, 15]. As a consequence, 20–25 W is commonly thought of as a laser power to be used with low risk of thermal injury within the pelvicalyceal system [16].

However, it is well recognised that irrigation fluid flow rate also significantly influences the temperature rise during laser activation [11, 12, 13, 17, 18]. Therefore, if the irrigation fluid flow rate is increased or more consistent it may better mitigate a laser‐induced temperature rise enabling a lower risk profile for use of laser settings of higher power.

With the advent of flexible and navigable access sheaths (FANS), which cross the PUJ to sit within the pelvicalyceal system, increasing irrigation flow rate does not result as strongly with elevated intrarenal pressures (IRPs) because of the low outflow resistance and negative suction pressure [19]. However, maintaining consistent higher flow rates may be difficult with gravity driven or pressure‐cuff bags that require manual intervention to manage the pressure. To tackle this problem, there have been recent advances in endourology irrigation with fluid management systems (FMS) based on an automated pump [20]. There are now several FANS and ureteroscopes that have IRP monitoring and integration with an automated FMS [21, 22, 23].

**Fig. 1:**
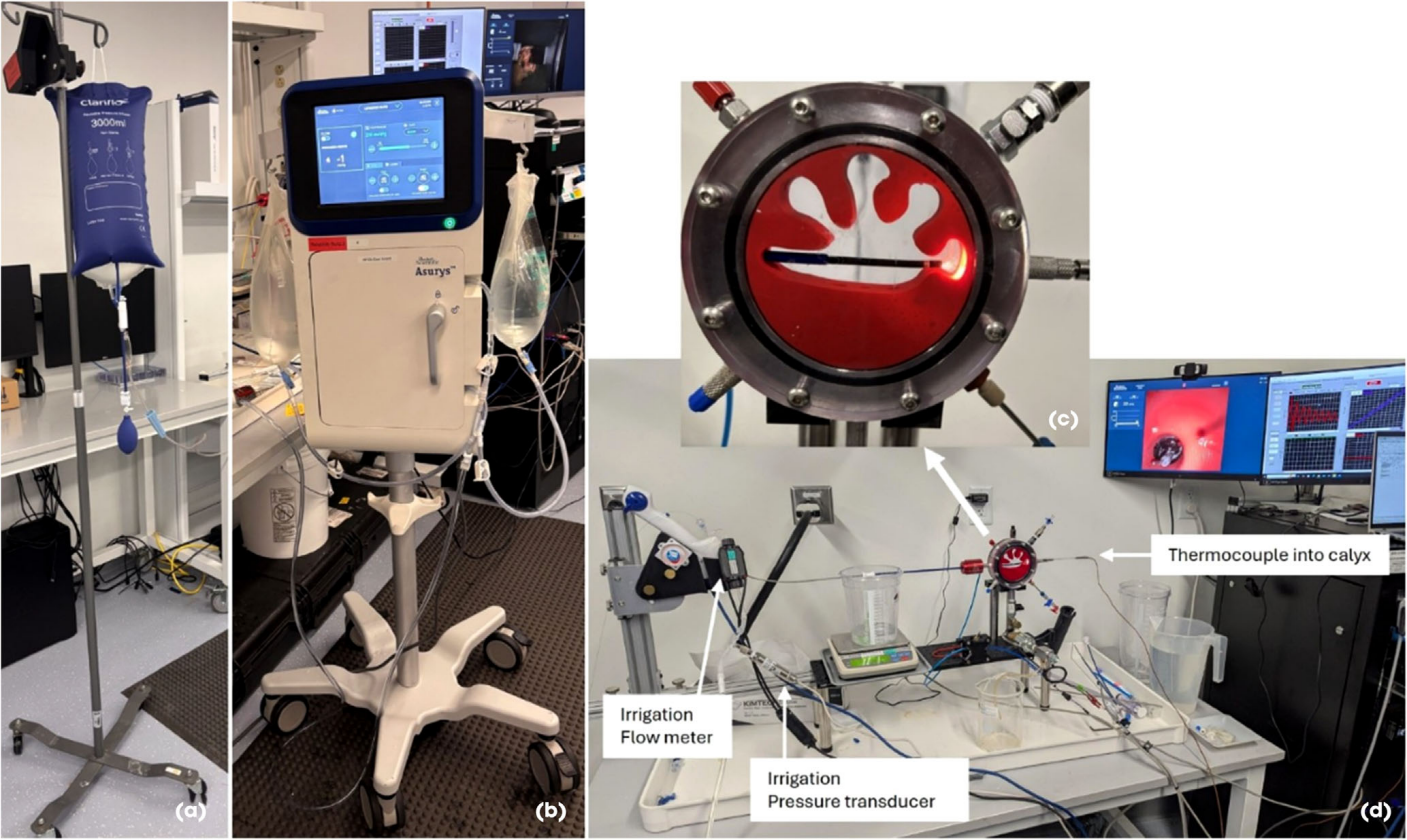
Research laboratory test setup photographs of **(a)** 3‐L saline bag in pressure cuff (pressure bag), **(b)** Asurys FMS, **(c)** three‐dimensional printed kidney calyx model, and **(d)** full Boston Scientific bench model ureteroscopy setup: a 9.5‐F LVE (Boston Scientific Corp.) ureteroscope was placed within a renal calyx of the benchtop model. An 11/13‐F ureteric access sheath (Navigator™ HD, Boston Scientific Corp.) was within the renal pelvis. A 200‐μm laser fibre (MOSES™ 200 D/F/L, Boston Scientific Corp.) was introduced through the ureteroscope working channel and fired in the centre of the calyx.

The Asurys™ FMS (Boston Scientific Corp., Marlborough, MA, USA) evaluated in this study is specifically designed to work with LithoVue™ Elite (LVE; Boston Scientific Corp.) single‐use digital flexible ureteroscope with real‐time IRP monitoring (Fig. 1). Together this system is designed to autoregulate irrigant flow rate based on IRP data and physician‐selected IRP settings [24]. This system also has a feature designed to maintain expected fluid flow when an instrument occupies the LVE ureteroscope working channel.

This study performed bench testing of this concept FMS vs a traditional pressure bag irrigation to evaluate their ability to maintain higher or more consistent flow rates and the consequent temperature increases from laser activation in a benchtop model. This was used to validate a mathematical model, which was then used to predict the ‘thermal dose’ imparted on the renal calyx from laser activation as a function of laser power (W) and irrigation fluid flow rate.

## Materials and Methods

### Bench‐Top Experiments

A 9.5‐F LVE ureteroscope system was placed, within an 11/13‐F Navigator™ HD ureteric access sheath (UAS; Boston Scientific Corp.) into a calyx of a benchtop kidney model at room temperature (Fig. 1). The UAS was placed within the renal pelvis and ureteroscope within the renal calyx. The model kidney was closed with no outflow ‘down the ureter’ outside of the UAS. Room‐temperature irrigation was provided by either a 3 L saline bag with a manual inflatable cuff irrigation‐bag (pressure bag; clariflo™ 3000 mL reusable pressure infuser, Tapmedic LLC, Jacksonville, FL, USA) pressurised to 200 mmHg (measured via the irrigation tubing inline pressure sensor PX409 pressure transducer, OMEGA Engineering, Inc., Norwalk, CT, USA) or the Asurys FMS set to 200 mmHg with a flow limiter IRP set‐point at 40 mmHg (this set‐point enables irrigation modulation based on real time LVE pressure sensor measurement).

Both irrigation setups were pressurised and left to run for 10 min prior to lasing. Throughout the 10 min live irrigation pressures (measured via the irrigation tubing inline pressure sensor – PX409, OMEGA), flow rates (measured via FD‐XC8R2 and FD‐XS8 flow meter, Keyence Corp., Itasca, IL, USA) and IRPs were recorded in both irrigation system test arms (via LVE single‐use digital flexible ureteroscope system with IRP monitoring).

After 10 min, a 200‐μm laser fibre (MOSES™ 200 D/F/L, Boston Scientific Corp.) was fired continuously in the centre of the calyx at 20 (20 Hz, 1 J), 40 (40 Hz, 1 J) or 60 W (60 Hz, 1 J) for 90 s with MOSES distance mode activated. Intra‐calyceal temperatures in the bench model were recorded and reported for 10 s prior to laser activation, the 90 s of laser activation and for 20 s after laser activation (via P120 laser with MOSES™ 2.0, Boston Scientific Corp.; measured in benchtop model with fine‐tip TJ probe thermocouple inserted into calyx, TJFT72 OMEGA).

Both arms were repeated five times, each with a different LVE ureteroscope, UAS and laser fibre.

### Mathematical Model

Our mathematical model used to predict renal calyx temperature during laser activation has been described previously [17]. The key model parameters have been outlined in Appendix S1.

First, the mathematical model was used to predict the change in calyx temperature (starting at measured room temperature) when activating the laser at 20, 40 and 60 W in the silicone model calyx as a function of different flow rates. Calyx temperature was then used to calculate the equivalent minutes (eq min) at 43 C (CEM43) ‘t43 equivalent’ thermal dose of laser activation.

Second, the mathematical model was used to predict calyx temperature as a function of flow rate in an *in vivo* (porcine) kidney (starting at body temperature of 37°C). Calyx temperature was used to calculate the ‘t43 equivalent’ thermal dose of laser activation in *in vivo* conditions.

### The t43 Calculations

Thermal dose was calculated from the empirical bench test dataset via the Sapareto and Dewey [25] validated t43 equivalent methodology [26]. The ‘thermal dose’ imparted on the renal tissue is determined by both temperature and exposure time [12, 25]. It is reported in eq min at 43°C and represents cumulative temperature exposure [12, 25].

Cellular thermal injury (protein denaturation) occurs when temperature reaches or exceeds 43°C for a sufficient length of time. A standardised threshold for potential injury and cell death is 120 eq min at 43°C, although there is some variability [12, 18, 25, 26]. This means that the threshold of tissue injury is reached after 120 min of exposure at 43°C. Temperatures <43°C would never reach this threshold but increasing temperatures above 43°C leads to an exponential decrease in the time to reach the threshold. For instance, at 44°C the threshold is reached in 60 min; 45°C in 30 min; 50°C in 1 min; 56°C in <1 s [12].

The CEM43 thermal dose was calculated with the following equation (which is based on the Sapareto and Dewey [25] method):

$$\mathrm{CEM}43=\begin{array}{l} \text{Duration of laser activation}\\ \times R^{\left(43–\text{maximum steady}-\text{state temperature}\right)} \end{array}$$

- The ‘duration of laser activation until threshold is breached’ was consequently calculated by:

$$\text{Duration of laser activation}=120\;\min/R^{\left(43–\text{maximum steady}-\text{state temperature}\right)}$$

In both equations: *R* = 0.25 if maximum temperature <43; *R* = 0.5 if maximum temperature >43.

- The ‘maximum temperature’ was derived from the model prediction of calyceal temperature starting at body temperature. This approach, using the maximum rather than the average temperature over 90 s of laser activation, differs from the Sapareto and Dewey [25] method. It yields a higher ‘t43 equivalent’ thermal dose and represents the most conservative method of estimation, allowing us to estimate the duration of continuous laser activation until thermal threshold is exceeded.

## Results

**Fig. 2:**
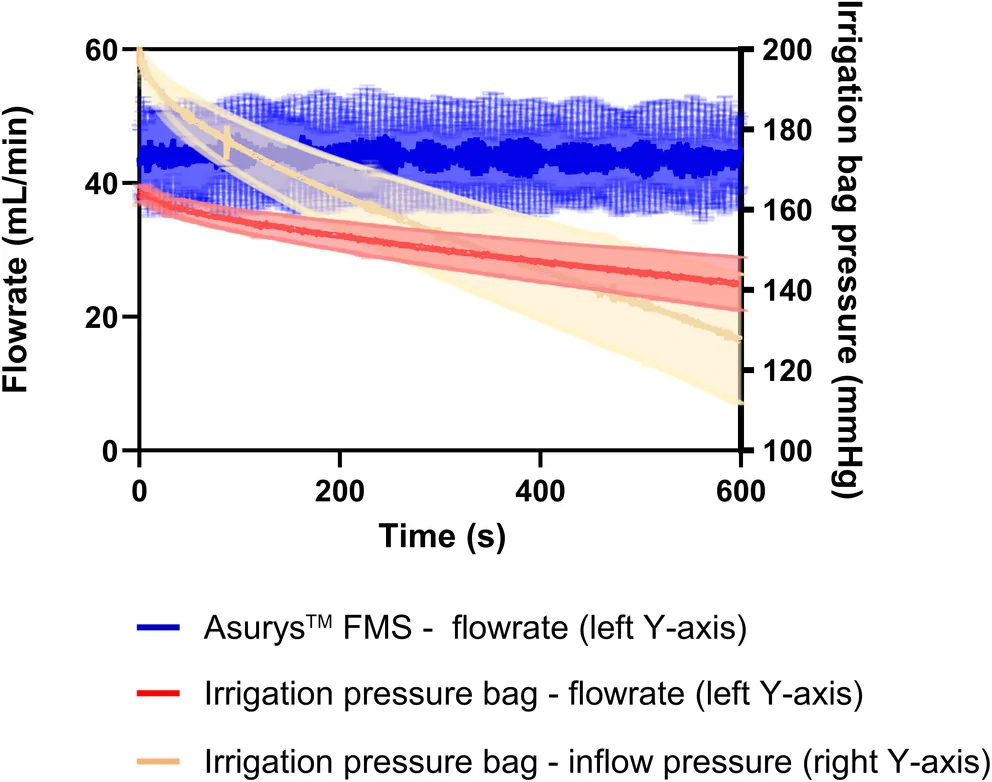
Irrigation pressure‐bag inflow pressure (pink), irrigation pressure‐bag flow rate (red) and Asurys^TM^ FMS flow rate (blue) when left for 10 min after devices were pressured to 200 mmHg. Over 10 min, irrigation bag pressure decreased from 200 mmHg to 128 mmHg (range: 105–150 mmHg), reducing flow rates from 38 mL/min to 25 mL/min (35% decrease). Concept‐FMS maintains a consistent flow rate of ~45 mL/min throughout.

The pressure bag mean (SD) irrigation pressure at the start of the experiment was 201 (0.8) mmHg and the flow rate was 38 (1) mL/min (with the bag specifically pressurised to the target 200 mmHg irrigation pressure as measured on the inflow line pressure transducer). After 10 min of fluid flow and no management of the irrigation, the mean (SD) pressure bag irrigation pressure decreased to 128 (14) mmHg with a flow rate of 25 (4) mL/min. This represents an average reduction of 35% and 36% in irrigation pressure and flow rates, respectively (Fig. 2).

The initial mean (SD) flow rates for the Asurys FMS was 44 (4) mL/min at a 200 mmHg irrigation setpoint with the flow modulated to meet the flow limiter IRP setpoint of 40 mmHg. This output flow rate measured from the Asurys FMS is a function of three system features working together: the 200 mmHg irrigation setpoint, the activation of the feature to compensate for a tool in the working channel (laser fibre presence), and modulation to maintain the IRP setpoint of 40 mmHg. After 10 min, the Asurys FMS flow rate was maintained at a mean (SD) of 45 (5) mL/min. The mean Asurys FMS flow rate after 10 min was 76% higher than with the mean pressure‐bag flow rate after the 10 min of fluid flow. The IRP measured in the calyx was 30 mmHg or less in both groups in all experiments.

At the start of 90 s of laser activation (10 min after irrigation devices were initially pressurised), the mean (SD) starting temperature of the Asurys FMS and pressure‐bag groups was 22.3 (0.6)°C vs 21.7 (0.5)°C (*P* = 0.03, *t*‐test).

**Fig. 3:**
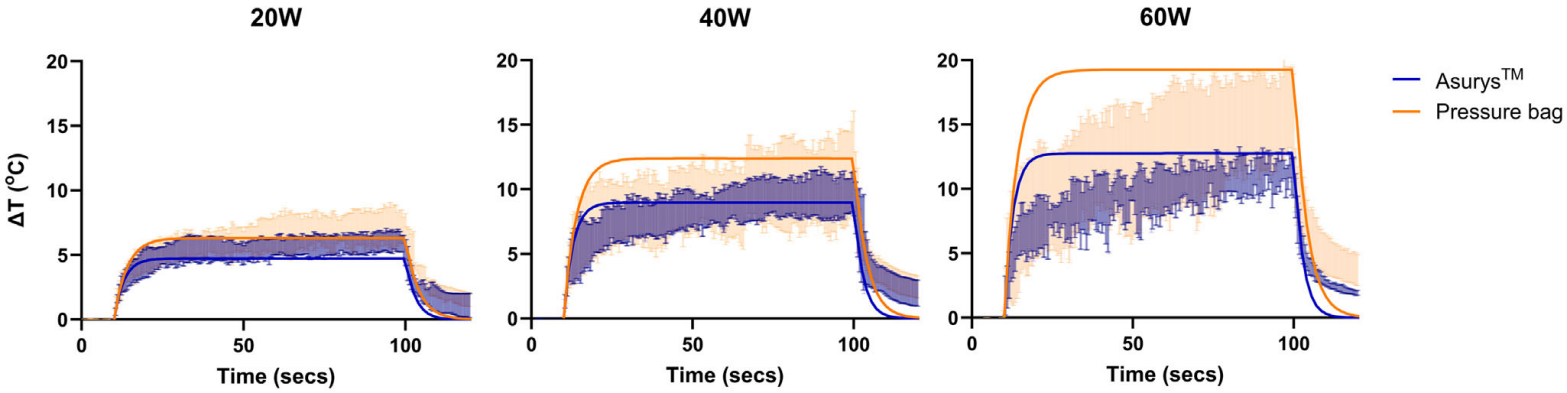
Benchtop empirical vs modelled temperature change (from starting room temperature) with activating a 20/40/60 W laser for 90 s with pressure bag (inflow flow rate at 25 mL/min) vs Asurys FMS (inflow flow rate at 45 mL/min) driven irrigation flow in a benchtop model. Error bars (95% CI) represent benchtop data; Solid lines represent model prediction.

Benchtop data showed that laser activation increased calyx temperature in all groups (Fig. 3). Increasing laser power (W) resulted in higher calyx temperature and thermal dose. Higher flow rates, generated from the Asurys FMS vs pressure bag, resulted in lower calyx temperatures and thermal doses at 20, 40 and 60 W (Fig. 3 and Table 1). Empirical mean (SD) temperature rise (for five test replicates) with pressure bag vs FMS irrigation was: at 20 W 7.9 (1.0)°C vs 6.4 (0.6)°C (*P* = 0.02); at 40 W 12.8 (3.2)°C vs 10.3 (1.7)°C (*P* = 0.15); at 60 W 17 (3.4)°C vs 12.4 (1.2)°C (*P* = 0.02).

**Table 1:** First, in a benchtop silicone model: empirical vs modelled temperature rises and ‘t43 equivalent’ thermal doses with varying laser powers and irrigation systems. Second, in *in vivo* conditions: mathematically modelled temperature and ‘t43 equivalent’ thermal dose with laser activation for 90 s.

Laser power, W	Irrigation system	Empirical temperature increase after 90 s laser activation in a benchtop model, °C	Modelled temperature increase after 90 s laser activation in a benchtop model, °C	Empirical thermal dose (t43 eq min) with 90 s of laser activation in a benchtop model, CEM43	Modelled thermal dose (t43 eq min) with 90 s of laser activation in a benchtop model, CEM43	Modelled *in vivo* temperature after 90 s of laser activation, °C	Modelled *in vivo* thermal dose (t43 eq min) with 90 s of laser activation, CEM43	Modelled *in vivo* duration of laser activation until thermal threshold is exceeded, min
20	Pressure bag	7.9	6.3	7	2	33.4	3 × 10^−6^	>120
20	Asurys FMS	6.4	4.7	2	0.4	31	9 × 10^−8^	>120
40	Pressure bag	12.8	12.4	947	127	39.6	1 × 10^−2^	>120
40	Asurys FMS	10.3	8.9	48	12	35.1	3 × 10^−5^	>120
60*	Pressure bag	17	19.3	1.6 × 104	1.5 × 10^4^	46.7	19	9
60	Asurys FMS	12.4	12.8	167	164	38.8	4 × 10^−3^	>120

When the Asurys FMS was used for irrigation, the laser could theoretically be activated for >120 min at all laser powers without the t43 thermal dose being exceeded in a benchtop model. With a pressure bag, the t43 thermal dose would be exceeded after 9 min with 60 W (*).

There was reasonable agreement between the empirical data and the mathematical model in predicting temperature rise (Fig. 3) and thermal dose (Table 1) as a function of laser power and irrigant flow rate. In the 40 and 60 W laser power scenarios, the mathematical model predicted the final calyx temperature (at 100 s) within the 95% CI range of the empirical data. At 20 W there was a discrepancy of 1.7°C between the mathematical model prediction and the empirical data. This is comparable to the empirical instrumentation measurement tolerances. The model over‐predicted the rapidity of temperature increase vs empirical data. This is because the mathematical model assumed a closed calyx volume of 3 mL, but in the benchtop model, fluid in the (3 mL) calyx mixed with the fluid in the rest of the pelvicalyceal collecting system. These models provide an example within the potential clinically relevant *in vivo* geometry range in which laser‐induced temperature increase would be of interest.

**Fig. 4:**
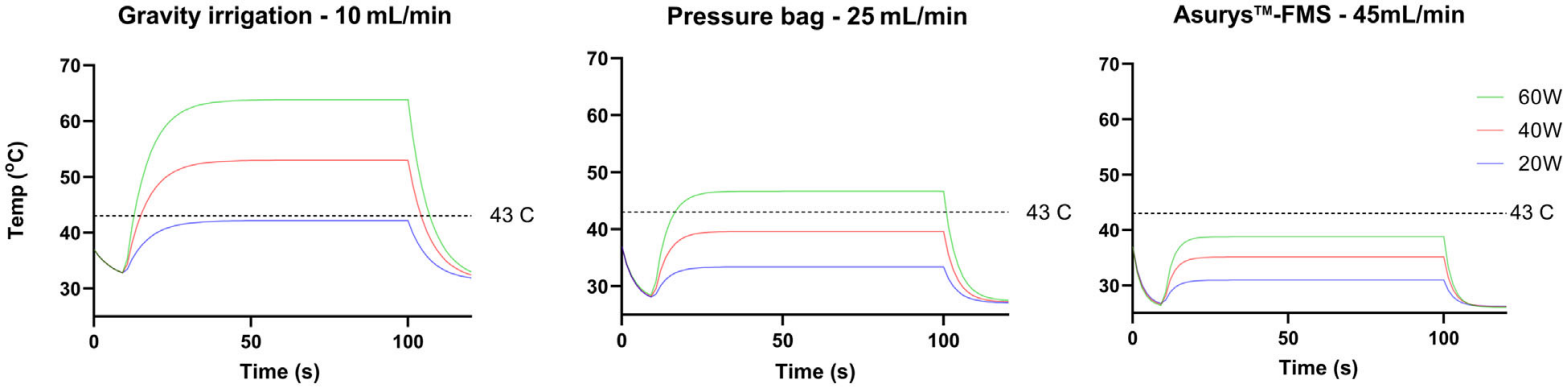
Mathematically modelled *in vivo* (porcine) renal calyx temperature when activating a laser at 20/40/60 W. Flow rates of 10 mL/min (approximate from gravity bag – left) vs 25 mL/min (pressure bag – middle) vs 45 mL/min (Asurys FMS – right). Starting temperature of 37°C, 10 s of irrigation (irrigation fluid 21°C) results in cooling, laser activation for 90 s then results in calyceal heating. Temperature remains <43°C temperature threshold at all laser powers with an irrigation flow rate of 45 mL/min.

Modelling *in vivo* (porcine) calyx temperature (Fig. 4) predicts that with the Asurys^TM^ FMS driven flow (45 mL/min), the steady‐state calyx temperature is maintained under the 43°C threshold when activating a 20, 40 and 60 W laser. With pressure‐bag driven flow (25 mL/min), 60 W laser activation results in a steady‐state temperature of 47°C (breaching the 43°C threshold), while a steady‐state calyx temperature <43°C was maintained when activating the 20 and 40 W lasers.

Out of the configurations tested, only the pressure‐bag group (25 mL/min) at 60 W was estimated to breach the thermal dose threshold (CEM43) (Table 1). In this group, laser activation for 90 s is predicted to generate an *in vivo* (given the assumptions/limitations of the translation) CEM43 of 19.2 min – equivalent to heating the calyx at 43°C for 19.2 min. The thermal threshold (equivalent to heating the calyx at 43°C for 120 min) would be breached by laser activation for 9.4 min, indicating that irrigation systems delivering this lower flow rate could be at higher risk for thermal injury.

For all other experimental groups (pressure bag at 20 and 40 W; FMS at 20, 40, and 60 W) the thermal dose threshold is not estimated to be breached, which reflects the potential for continuous laser activation for >120 min at these settings and flow rate combinations with a lower risk for thermal injury.

**Fig. 5:**
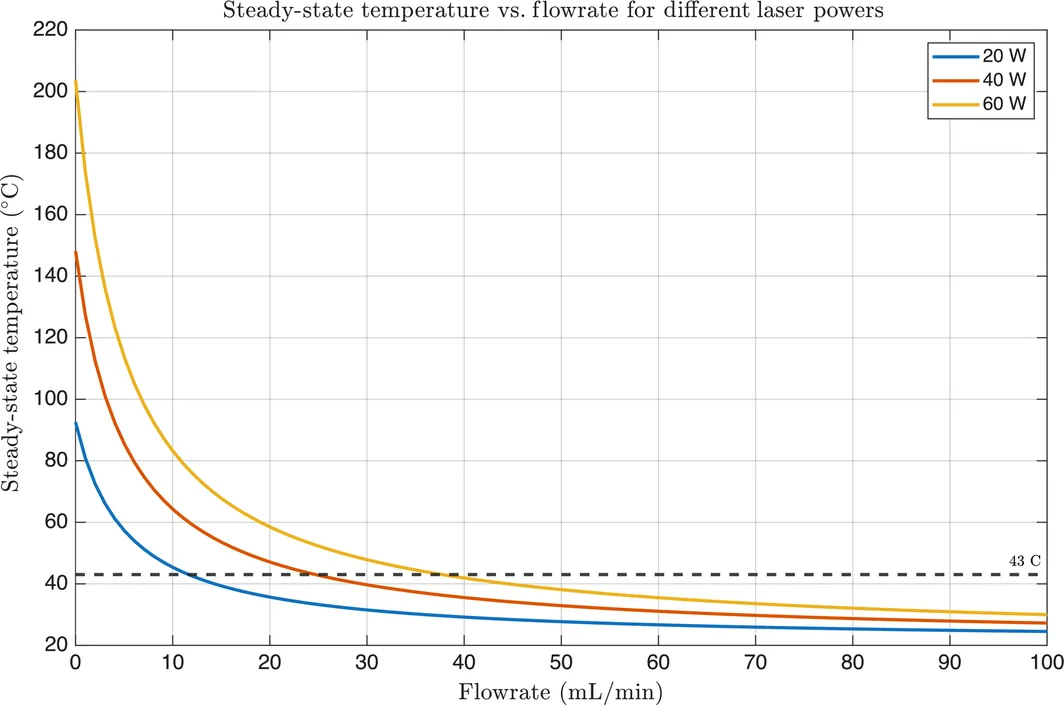
Mathematically modelled ‘steady‐state’ calyx temperature vs flow rate for different laser powers in an *in vivo* (porcine) kidney. For reference, gravity‐driven irrigation flow rate is ~10 mL/min.

By comparison, with irrigation flow rate at 10 mL/min (approximately the flow with 100 cm gravity‐driven irrigation, which is equivalent to 74 mmHg irrigation pressure), modelling suggests that the thermal dose could already be breached when lasing at just 20 W (Fig. 5) [27].

## Discussion

The findings from this study support trends between laser power, irrigation rate and thermal dose. Our results demonstrate, in a bench model, that the Asurys^TM^ FMS can automatically maintain higher consistent flow rates at an IRP <30 mmHg. While irrigation with a manual inflatable cuff irrigation‐bag without manual intervention cannot maintain consistent pressure and results in decreasing flow rates over time.

Irrigation fluid flow rate is inherently unstable in manual pressure bags due to loss of pressure as the irrigation fluid reduces (Boyles Law). Consequently, automated FMSs have been developed for clinical use that use a digitally‐controlled peristaltic pump, which are inherently more stable. The Asurys^TM^ FMS evaluated here specifically works together with LVE Single‐Use Digital Flexible Ureteroscope with real‐time IRP monitoring. This system has the ability to optimise flow rate based on user settings along with the modulation of flow to be maintained with a tool in the working channel. The results shown here support the flow rates useful in mitigating thermal risk.

The higher consistent flow rates from the Asurys^TM^ FMS in the bench evaluation mitigated laser‐induced temperature increases at all laser power settings compared to the pressure bag. Figure 4 shows the results of the physics‐based mathematical model thermal dose predictions *in vivo*. This suggests that laser can be activated with calyx temperature maintained at ≤43°C with the following flow rate and laser power setting combinations: 20 W (20 Hz, 1 J) with 10 mL/min (approximate flow rates with gravity); 40 W (40 W, 1 J) with 25 mL/min; and 60 W (60 Hz, 1 J) with 45 mL/min.

These findings are consistent with other benchtop, *in vivo* and *ex vivo* porcine studies that suggest that increasing irrigant flow rate can further mitigate thermal dose, lower thermal injury risk, and potentially allow higher laser power to be used [11, 12, 13, 14, 17, 18, 28, 29]. An *in vivo* porcine experiment demonstrated that room temperature saline irrigation at 40 mL/min prevents substantial temperature elevation in the ureter even with up to 60 W laser power [12].

Our study is novel in demonstrating how automated FMSs may facilitate such flow rates to better manage risk of thermal injury, while maintaining low IRPs. For a pressure bag to continuously maintain these higher flow rates it would require frequent manual re‐inflation. A FMS can digitally automate maintaining these higher flow rates without this irrigation management.

Our study has several limitations. Most notably, the empirical data were derived from a benchtop model maintained at room temperature, which does not replicate the full physiological complexity of the *in vivo* kidney. The model was intentionally simplified to illustrate the relationship between irrigation flow rate, laser power, and temperature rise.

The other critical difference between the benchtop and *in vivo* settings is the heat transfer coefficient—a parameter describing how efficiently heat dissipates through the kidney wall or model boundary. For reference, the heat transfer coefficient of an *in vivo* porcine kidney is ~0.36 W/°C, compared to the 1.15 W/°C used in our benchtop simulations (Appendix S1). This heat transfer coefficient has a substantial impact on steady‐state temperatures. As such, while the benchtop data provided useful trends and validation of the mathematical model, all results reported here are limited in estimating the thermal dose (CEMt43) to *in vivo* clinically relevant conditions. However, reassuringly, the model demonstrates reasonable predictive agreement with published *in vivo* porcine data [11, 12, 13, 14].

The *in vivo* porcine kidney heat transfer coefficient may not be a perfect proxy for *in vivo* human kidney due to differences in: perirenal fat; compliance; extra‐renal anatomy; and pelvicalyceal anatomy. As such these benchtop findings need to be validated with *in vivo* human data – currently there are no reported measurements for human *in vivo* kidney heat transfer coefficients.

Second, we chose to simplify the bench testing, by activating the laser into an empty calyx rather than performing lithotripsy of a renal stone. This simplification was in order to minimise variability between experiments but may preclude an important determining factor of calyx temperature if laser energy is absorbed by the stone. However, benchtop data suggests that the inclusion of stone lithotripsy would make little difference to fluid temperature [30]. In addition, the model had a low outflow resistance (as a consequence of the large access sheath) allowing higher flow rates. In clinical practice, sheathless ureteroscopy, smaller sheaths or sheath occlusions may increase outflow resistance. Flow rate from automated systems based on managing IRP measurements may need to balance these two risks when selecting irrigation setpoints, reducing flow rate. As demonstrated in Fig. 5, decreasing flow rate exponentially increases calyx temperature. Therefore consistent low outflow resistance is necessary to facilitate the flow rates to use higher power, which could also vary intraoperatively with debris build up in the sheath.

Third, there was reasonable agreement between the mathematical model and the empirical bench data. This mathematical model has been previously validated with several *in vitro* experiments, showing excellent agreement with uniform spheres as model calyces [17]. However, the bench model used in this experiment reflects a more clinically relevant geometry of a pelvicalyceal system (with calyces and a renal pelvis) allowing representative fluid mixing within the kidney. The mathematical model does not perfectly account for the mixing of fluid between calyces and renal pelvis but rather is fit for an ‘effective volume’ to simplify.

Fourth, this study demonstrates the capability of the LVE ureteroscope connected to the Asurys FMS to modulate flow based on the IRP measured by the scope pressure sensor, which is limited to the specified pressure sensor tolerance (specifications per LVE with pressure monitoring, single‐use digital flexible ureteroscope – IFU 51227270). However, methodologically by using the same sensor for the intervention (flow rate) and for measuring the secondary outcome, this does potentially introduce a detection bias, but the study utilised the same scope pressure sensor setup for IRP measurement for both irrigation test arms. Temperature and flow rate were independently measured by additional instrumentation and not affected.

This benchtop experiment has been performed using a holmium:yttrium‐aluminium‐garnet (Ho:YAG) laser; however, it is worth noting that the thulium fibre laser and Ho:YAG have the same thermal profile at same laser settings (including energy and frequency) [31, 32].

Data reported here aligns with *in vivo* porcine preclinical experiments demonstrating that irrigation flow rates of 15 and 40 mL/min can manage the temperature increase in a bench model when activating a 20 and 60 W laser respectively [11, 12, 13, 14]. However, further *in vivo* preclinical and clinical research is needed to understand if these findings translate into *in vivo* human kidneys.

## Conclusions

Irrigation fluid flow rate significantly influences calyceal temperature during laser activation. Higher flow rates mitigate temperature rise. Manual inflatable cuff irrigation‐bags rapidly deflate with a subsequent decline in flow rate. By contrast, flow rates remained relatively stable and high while using a FMS.

The use of a FMS, with an automated and consistent high flow rate, may allow higher laser power (W) to be used while maintaining a lower calyx temperature. Higher laser power may enable faster lithotripsy and allow larger renal stones to be managed by retrograde intrarenal surgery.

## Disclosure of Interests

Richard Menzies‐Wilson, Ben Turney, Leilane Glienke, William W. Roberts are recipients of Boston Scientific Corporation Research grant. Candace Rhodes and Jessica Williams are employees of Boston Scientific Corporation.

## Disclosures

The testing was performed by or on behalf of Boston Scientific Corporation. Data on file. This study was funded by Boston Scientific Corporation. Candace Rhodes and Jessica Williams are employees of the Boston Scientific Corporation The Boston Scientific Corporation‐concept FMS used in this study was a concept device further developed into the Asurys™ FMS, which is pending 510(k) clearance and not yet available for sale in the USA and is not Conformité Européenne (CE) marked nor available for sale in the European Union. The data from the concept device may not necessarily represent the final device intended to be sold (not yet available at time of study). Bench testing results may not necessarily be indicative of clinical performance. The Boston Scientific Corporation acquired the global surgical business of Lumenis Ltd. Some registered names of products manufactured and sold by Boston Scientific may contain the term ‘Lumenis’. Lumenis is a registered trademark of Lumenis.

## Supporting information


**Appendix S1.** Mathematical model pelvicalyceal temperature as a function of flow rate and laser power.
**Fig. S1.** Steady state calyx temperature vs heat transfer coefficient when firing a laser of different powers in a 3 mL calyx.

## Data Availability

Research data are not shared.
