# Supplementary material for: Automated irrigation systems in mitigating laser‐induced temperature during simulated ureteroscopy
Source: BJU Int. 2026 Jul 7;138(4):650–8. doi: 10.1111/bju.70364 (PMC13550810; doi:10.1111/bju.70364)
Supplement: Supplementary file 1 [file BJU-138-650-s001.docx]

Appendix S1

**Mathematical model pelvicalyceal temperature as a function of flow rate and laser power**

The key assumptions in the mathematical model were:

Calyx Volume - 3mL (Figure 3 and Figure 4); Figure 5 is independent of volume

Irrigant fluid temperature 21C (Figure 4 & 5); Data starting temperature (Figure 3)

Boundary temperature - 37C (Figure 4 & 5); Data starting temperature (Figure 3)

Heat transfer coefficient - 0.36 Watts/C (Figure 4 & 5); 1.15 Watts/C (Figure 3)

density - 1000 kg/m 3

specific heat capacity of irrigation fluid - 4179.6 J/kgC

The heat transfer coefficient describes the transfer of heat through the material containing the

fluid. The higher the heat transfer coefficient, the faster the transfer of heat, through the material, out of the volume of fluid. Independent of other variables the heat transfer coefficient has a significant effect on the steady state temperature (Figure A).

The heat transfer coefficient varies significantly between materials. Consequently, when predicting in-vivo kidney temperatures it is important to use a physiological heat transfer co-efficient.

The heat transfer coefficient of 0.36Watts/C were calculated from two separate in-vivo porcine

kidney experiments performed by two of the authors (LG & WR). In these, a laser was activated within a calyx of an in-vivo pig under general anaesthesia. By controlling all variables and recording the resulting temperature, the heat transfer coefficient could be calculated. In two

separate experiments the heat transfer co-efficient was 0.35Watts/C and 0.36Watts/C.

Figure S1: Steady state calyx temperature vs. heat transfer co-efficient when firing a laser of different powers in a 3mL calyx.
